# Errata

**Published:** 1873-12

**Authors:** 


Errata.—On page 406, current volume, fifth line, for *digestion* read *deglutition*.

On page 407, eleventh line, for *other* read *proper*.

On page 419, in caption, for *No.* 9 read *No.* 8.

Subscribers will please turn back and make these corrections.

